# The prevalence of *BRCA1/2* mutations of triple-negative breast cancer patients in Xinjiang multiple ethnic region of China

**DOI:** 10.1186/2047-783X-19-35

**Published:** 2014-06-25

**Authors:** Yong Tao Li, Duo Ni, Liang Yang, Qian Zhao, Jiang Hua Ou

**Affiliations:** 1 Department of Breast Surgery, Tumor Hospital, Xinjiang Medical University, 789 East Suzhou Street, Urumqi 830011, China

**Keywords:** *BRCA1/2*, breast Cancer, triple-negative, genetic testing

## Abstract

**Background:**

The screening of *BRCA1* and *BRCA2* mutations is now an established component of risk evaluation and management of familial breast cancer, early-onset breast cancer and bilateral breast cancer patients. There is still some controversy about whether this screening should be done in triple-negative breast cancers. Therefore, we evaluated the *BRCA* mutation prevalence in patients with triple-negative breast cancer in a multi-ethnic region of China.

**Methods:**

A total 96 women who were diagnosed with triple-negative breast cancer in the Xinjiang region of China were enrolled in this study. *BRCA1* and *BRCA2* screening was performed by polymerase chain reaction-denaturing high-performance liquid chromatography (PCR-DHPLC) sequencing analysis. All mutations were confirmed with direct sequencing.

**Results:**

The prevalence of a *BRCA1/2* germline mutation was about 25% (24/96) in the Xinjiang region of China. Among 35 selected cases with a family history and/or bilateral breast cancers, the *BRCA1/2* mutation prevalence was 25.7% (9/35). Of the remaining 61 patients with unselected triple-negative breast cancer, the *BRCA1/2* mutation prevalence was 24.6% (15/61), and all 15 individuals with these mutations were premenopausal patients.

**Conclusions:**

These results suggest that premenopausal women with triple-negative breast cancer may be candidates for genetic testing for *BRCA1/2* in the Xinjiang region of China, even in the absence of a family history or bilateral breast cancer.

## Background

The two major contributors to hereditary breast cancer are the breast cancer susceptibility gene 1 (*BRCA1*) and *BRCA2*[1]. Genetic testing for *BRCA1* and *BRCA2* mutations has been established throughout North America and most of Europe. Not all women are candidates for testing; in general, testing is only necessary for women who have a familial breast cancer, early-onset breast cancer or bilateral breast cancer.

China is a multi-ethnic country, but the majority of the population belongs to the Han ethnic group. Chinese breast cancer patients on average were younger than patients in America and Europe by about 10 years old, and premenopausal patients accounted for the majority [2]. The prevalence of *BRCA1* and *BRCA2* mutations among the Chinese Han population was less than 20%, mutation of *BRCA2* gene was higher than that of *BRCA1*[3,4], and this is significantly different than for America and Europe populations [5].

To date, only a few studies have been reported on *BRCA*-associated breast cancer in China; all the studies involved mainly ethnic Han Chinese. Our previous study showed that the prevalence of the *BRCA1/2* germline mutation was about 7.6% (6/79) in the Xinjiang multi-ethnic region of China. Among six of these *BRCA*-related tumors, three *BRCA1*- and one *BRCA2*-associated tumors were in estrogen receptor (ER), progesterone receptor (PR) and human epidermal growth factor receptor 2 (HER-2) triple-negative status [6].

Triple-negative breast cancer (TNBC) describes a subgroup of tumors that lack expression of ER, PR and HER-2. Due to the lack of effective targets for endocrinal and molecular treatment, it is considered a type of high risk breast cancer, and the prognosis is poor. Overall, TNBC accounts for about 15% [7,8] of all breast cancers, but occurs more frequently in younger women and is the predominant subtype in individuals with a germline *BRCA1* mutation [9]. Therefore, we have evaluated the *BRCA* mutation frequency and the implications for the clinical practice of undertaking genetic testing in women with TNBC.

## Methods

### Cases

All patient cases were recruited from Xinjiang, China. They had a definite pathological diagnosis of breast cancer and received the standard treatment in our hospital during the period 2005 to 2013. At the beginning of this period, women diagnosed with breast cancer who had family history (FH), early onset, or bilateral breast cancer (BI-BC) were chosen for study. As the research developed, we found that the majority of patients with the *BRCA1/2* germline mutation were in triple-negative status. Therefore, TNBC patients were included in our study. To date, a total of 214 patients were submitted for evaluation, of which 96 were TNBC.

The study was approved by the Ethics Committee (IRB approval number: XJYD1320) of the Cancer Hospital of Xinjiang Medical University, and informed consent was obtained from each participant.

### Triple-negative breast cancer confirmed

ER, PR and HER-2 status were confirmed in a histopathology report of the tumor samples. HER-2 was regarded as negative when scored as 0 or 1 by immunohistochemistry (IHC) and/or when there was non-amplification of HER-2 mRNA by fluorescent *in situ* hybridization (FISH).

### BRCA1/2 mutation analysis

Genomic DNA was isolated from 5 mL of peripheral blood and stored in 10 mM Tris (pH 8) EDTA at 4°C. Specific BRCA1 and BRCA2 coding regions and intron-exon boundaries, ranging from 206 to 639 bp in length, were amplified by polymerase chain reaction (PCR). Denaturing high-performance liquid chromatography (DHPLC) analysis was used to screen BRCA1 and BRCA2 mutations; all variants identified were confirmed by direct sequencing.

### Statistical analysis

To compare the BRCA1/2 mutation in unselected and selected TNBC, chi-square analysis was performed. A *P* value of <0.05 was considered statistically significant. The SPSS statistical software system version 16.0 (SPSS Inc., Chicago, USA) was used.

## Results

### Patients’ characteristics

A total of 96 women with TNBC were eligible for testing. Among them, 35 women were selected for study because they had FH and/or BI-BC (25 women had a FH of breast or ovarian cancer, 13 women were BI-BC, both a FH and BI-BC were identified in 3 women). The remaining 61 patients had unselected TNBC. The average age of the patients was 39.7 years old (range 24 to 64 years old).

### *BRCA1/2* mutations

The full results of all *BRCA1/2* deleterious mutations are given in Additional file 1: Table S1, all mutations were protein-truncating. Both the *BRCA1* and *BRCA2* mutation was identified in one patient with a family history, and she was 47 years old. *BRCA1/2* mutations were present in 24 out of 96 individuals with TNBC. The prevalence of the *BRCA1/2* germline mutation was about 25% (24/96), 18 in *BRCA1* and 7 in *BRCA2* (Table 1). The prevalence of *BRCA1* germline mutation was about 18.6% (18/96), and the prevalence of *BRCA2* germline mutation was about 7.3% (7/96).

**Table 1:** ***BRCA1 *** **and *BRCA2 *** **mutation prevalence in premenopausal/postmenopausal triple-negative breast cancer (TNBC) patients**

	**Number of cases**	***BRCA1 *** **mutations (%)**	***BRCA2 *** **mutations (%)**	**Total *BRCA1/2 *** **mutations (%)**
All	96	18 (18.6)	7 (7.3)	24 (25)^a^
Premenopausal	81	16 (19.8)	7 (8.6)	22 (27.2)^a^
Postmenopausal	15	2 (13.3)	0	2 (13.3)
All unselected TNBC	61	11 (18)	4 (6.6)	15 (24.6)
Premenopausal	55	11 (20)	4 (7.3)	15 (27.3)
Postmenopausal	6	0	0	0

^a^Both *BRCA1* and *BRCA2* mutations were identified in one patient with family history, and she was 47 years old.

Eighty-one of the 96 women were premenopausal patients, The *BRCA1/2* mutation prevalence was about 27.2% (22/81); among them, *BRCA1* mutation prevalence was about 19.8% (16/81), and *BRCA2* mutation prevalence was about 8.6% (7/81). The remaining 15 cases were postmenopausal patients, the *BRCA1/2* mutation prevalence was about 13.3% (2/15), the *BRCA1* mutation prevalence was about 13.3% (2/15), and *BRCA2* had no mutation. If we remove the 35 cases with family history and/or bilateral breast cancers from the analysis, a total of 15 (24.6%) *BRCA1/2* mutations were identified in the remaining 61 patients of unselected triple-negative breast cancer. All mutations in 15 individuals were premenopausal patients. Among the 15 patients, 11 (18%, 11/61) had *BRCA1* mutations, and 4 (6.6%, 4/61) had *BRCA2* mutations. Therefore, the prevalence of *BRCA1* mutations is greater than the prevalence of *BRCA2* in TNBC patients.

A total of 18 *BRCA1* mutations were identified; 13 of these mutations (72.2%) were in exons 10 and 11. Eight *BRCA2* mutations were identified; six mutations among them (75%) were in exons 10 and 11.

### Comparison of *BRCA1/2* mutation between unselected and selected triple-negative breast cancer

A total of 96 women with TNBC were analyzed: 35 cases were selected TNBC (25 women had FH of breast or ovarian cancer, 13 women had BI-BC, and 3 women had a FH and had BI-BC) and 61 cases were unselected TN cancers. A total of 7 (28%) mutations were identified in 25 patients who had a family history, 2 (15.4%) mutations were identified in 13 patients who had bilateral breast cancer, and 15 (24.6) mutations were identified in 61 patients who had unselected TNBC (Table 2). A comparison of the three kinds of patients by chi-square test showed no significant difference (*P* = 0.687).

**Table 2:** ***BRCA1/2 *** **mutation prevalence in the unselected and selected triple-negative breast cancer (TNBC) patients**

**Cases classification**	***BRCA1/2 *** **Mutation**		***χ***^**2**^	***p***
	**Yes (%)**	**No**		
Family history	7 (28)	18	0.752	0.687^a^
Bilateral breast cancer	2 (15.4)	11		
Triple-negative	15 (24.6)	46		

^a^There was no significant difference among the three kinds of patients (TNBC with family history, TN cancers with bilateralbreast cancer and single TNBC) (*P* >0.05).

A total of 9 (25.7%) *BRCA1* and *BRCA2* mutations were identified in 35 selected patients, and 15 (24.65) *BRCA1* and *BRCA2* mutations were identified in the 61 unselected patients (Table 3). It makes no difference in the prevalence ofTNBC whether the *BRCA1/2* mutation is selected or not. When all 96 TNBC were broken down into different age groups, *BRCA1* and *BRCA2* mutations were 25% (5/20) in unselected women with TNBC ≤ 35 years old and 33.3% (2/6) in women with a FH and/or BI-BC, TNBC and ≤ 35 years old.

**Table 3:** ***BRCA1/2 *** **mutation prevalence in unselected/selected triple-negative breast cancer (TNBC) patients**

	**Number of cases**	***BRCA1 *** **mutations (%)**	***BRCA2 *** **mutations (%)**	**Total *BRCA1/2 *** **mutations (%)**
All selected TN	35	7 (20)	3 (8.6)	9 (25.7)
< 35 years	6	1 (16.7)	1 (16.7)	2 (33.3)
≥ 35 years	29	6 (20.7)	2 (6.9)	7 (24.1)^a^
All unselected TN	61	11 (18)	4 (6.6)	15 (24.6)
< 35 years	20	4 (20)	1 (5)	5 (25)
≥ 35 years	41	7 (17.1)	3 (7.3)	10 (24.4)

Selection criteria: Selected = bilateral breast cancer and/or family history of TNBC; Unselected = TNBC.

^a^Both *BRCA1* and *BRCA2* mutations were identified in one patient with family history, and she was 47 years old.

## Discussion

These data support the position that premenopausal TNBC is an indicator that can be used to help to identify candidates for *BRCA1/2* mutation testing. *BRCA1/2* germline mutations are responsible for genetic predisposition and may increase the risk for breast and ovarian cancer [1]. The prevalence of *BRCA* mutations varies among different populations due to founder mutation effects and environmental factors [10,11]. Genetic cancer risk assessment guideline and genetic testing for breast cancer have become standard clinical management for selected patients in Western populations. But the situation in China is less managed. Only a few of studies reported *BRCA* mutations among breast cancer patients in China; the subjects were mainly familial breast cancer, early-onset breast cancer and bilateral breast cancer. Based on these studies, the *BRCA* mutation rate is generally less than 20% in China [3,4,12]. Our data show the prevalence of the *BRCA1/2* mutation in 96 cases of TNBC was 25%, close to the previous studies.

TNBC is a subgroup of breast tumors with poor prognosis. TNBCs are the predominant cancer subtype with a germline *BRCA* mutation. The prevalence of the *BRCA* mutation was 10 to 30% [13-15]. A total of 15 (24.6%) mutations were identified in 61 patients who had unselected TNBC (Table 3). The frequency of unselected TNBC is similar to the frequency of *BRCA1/2* mutations in individuals that were selected for inclusion because of a family history or bilateral breast cancer. Our study included samples of unselected TN cancers, which were primarily diagnosedin premenopause; only six cases were postmenopausal. All mutations were found in premenopausal patients. The prevalence of the *BRCA1* germline mutation was about 18.6% (18/96), and the prevalence of the *BRCA2* germline mutation was about 6.3% (6/96). Prevalence of *BRCA1* mutations is more common than *BRCA2* in TNBC patients, and *BRCA1* mutation is mainly associated with TNBC. All the *BRCA1* mutations are in TNBC cases with higher histological grades of invasive ductal carcinoma. These findings coincided with earlier findings for Asian populations, including Chinese [3,10-12], where *BRCA1*-mutated tumors conferred the following features: they were ER- or PR-negative and had a higher histological grade, but exhibited less medullary carcinoma compared to the Western population [16]. Whereas about 70% of the patients from this earlier study of TNBC in China were premenopausal [17], in our study, all *BRCA* mutations in TNBC were in premenopausal patients. TNBC with family history may influence the *BRCA* mutation; when the 96 TNBCs were broken down into different age groups, *BRCA1* and *BRCA2* mutations were 25% (5/20) in unselected women (≤ 35 years old) with TNBC and 33.3% (2/6) were in women (≤35 years old) with FH and/or BI-BC TNBC, respectively. These results are consistent with those reported in [18]. Although this study is not a large-scale investigation (only 96 patients), our results suggest that diagnosis of premenopausal TNBC would be a suitable threshold for *BRCA* testing. It is also consistent with simulation data, suggesting that this testing threshold would be a cost-effective strategy [19].

China is a developing country with multiple ethnic groups. Due to variations in the breast cancer prevalence, molecular subtypes and onset age among the different races and ethnicities, and because *BRCA* mutations are also different, caution must be exercised when following the clinical guidelines of the genetic and high familial risk assessment of breast cancer in the The National Comprehensive Cancer Network (NCCN) data. Most of those data come from Western populations, and in China, genetic testing should be modified to better assess the Chinese population. Our study only provides some preliminary information of *BRCA* mutation of TNBC in the multi-ethnic region of Xinjiang, China. Our dataset is relatively small, but we confirmed similar observations made by others. To date, no single study has been large or definitive in China for TNBC BRCA mutations, and therefore, it is important to consider the results of all studies in aggregate. More precise figures would be obtainable from larger, prospective studies. We also found a number of potential BRCA mutation hotspot regions in Xinjiang TNBC patients, such as the mutation mainly in exons 10 and 11, but our conclusions need further study and more data collection.

## Conclusions

Premenopausal women with TNBC may be candidates for genetic testing for *BRCA1/2* in the Xinjiang region of China, even in the absence of a family history or bilateral breast cancer.

## Abbreviations

BC: breast cancer; BI-BC: bilateral breast cancer; *BRCA1*: breast cancer susceptibility gene 1; *BRCA2*: breast cancer susceptibility gene 2; DHPLC: denaturing high-performance liquid chromatography; ER: estrogen receptor; FH: family history; FISH: fluorescent *in situ* hybridization; HER-2: human epidermal growth factor receptor 2; IHC: immunohistochemistry; OC: ovarian cancer; PCR-DHPLC: polymerase chain reaction-denaturing high-performance liquid chromatography; PR: progesterone receptor; TNBC: triple-negative breast cancer.

## Competing interests

The authors declare that they have no competing interests.

## Authors' contributions

YTL, DN, JHO conceived and designed the study and analyzed the data. YTL, LY, QZ, wrote the paper. All authors read and approved the final manuscript.

## Supplementary Material

Additional file 1: Table S1 BRCA1 and BRCA2 mutation in triple-negative breast cancer patients. Click here for file
