# Supplementary material for: The prevalence of BRCA1/2 mutations of triple-negative breast cancer patients in Xinjiang multiple ethnic region of China
Source: Eur J Med Res. 2014 Jun 25;19(1):35. doi: 10.1186/2047-783X-19-35 (PMC4076498; doi:10.1186/2047-783X-19-35)
Supplement: Additional file 1: Table S1 — BRCA1 and BRCA2 mutation in triple-negative breast cancer patients. [file 2047-783X-19-35-S1.doc]

Table 1

BRCA1 and BRCA2 mutation in triple-negative breast cancer patients

| Gene | Exon | Mutation | Mutation type | Age | Ethnic | Note |
| --- | --- | --- | --- | --- | --- | --- |
| BRCA1 | 10 | 3294delT | deletion | 41 | han | mother(OC) |
|  |  |  |  | 41 | han |  |
|  |  | 981_982del | deletion | 47 | hui | mother(BC) |
|  |  | 2566T>C | nonsynonymous | 48 | han |  |
|  |  | 3640G>T | nonsynonymous | 41 | han | mother and sister(BC) |
|  |  | 3607C>T | nonsynonymous | 32 | han | sister(BC) |
|  |  | 2572C>T | nonsynonymous | 30 | uyghur |  |
|  |  |  |  | 40 | uyghur |  |
|  |  |  |  | 33 | uyghur |  |
|  |  | 2952delT | deletion | 42 | han |  |
|  | 11 | 2073delA | deletion | 52 | han | mother(OC) |
|  |  | 2394C>T | nonsynonymous | 44 | han |  |
|  |  | 1934G>A | nonsynonymous | 51 | han | BI-BC |
|  | I-5 | 212+1G>T | splicing | 41 | kazakh |  |
|  | 19 | 5267_5268insC | insertion | 26 | uyghur |  |
|  | 23 | 5470_5477del | deletion | 48 | han | BI-BC |
|  |  |  |  | 36 | han |  |
|  | I-16 | 5105+1G>A | splicing | 34 | uyghur |  |
| BRCA2 | 10 | 1627A>T | nonsynonymous | 42 | han |  |
|  |  | 1545_1546del | deletion | 46 | uyghur | mother(BC) |
|  | 11 | 5682C>A | nonsynonymous | 47 | hui | mother(BC) |
|  |  | 2059_2063del | deletion | 34 | han | mother(BC and OC) |
|  |  | 6359C>G | nonsynonymous | 29 | han |  |
|  | 14 | 7178_7179delTG | deletion | 45 | hui |  |
|  | 27 | 9976A>T | nonsynonymous | 39 | uyghur |  |

Both of BRCA1 and BRCA2 mutations were identified in one patient with family history and she was 47 years old.
